# Short-term mortality prediction in children with gastrointestinal congenital anomalies using a random forest classifier

**DOI:** 10.1038/s41390-025-04378-2

**Published:** 2025-09-15

**Authors:** Andreea Madalina Serban

**Affiliations:** Carol Davila University of Medicine and Pharmacy, Bucharest, Romania

## Abstract

**Background:**

We aim to develop a predictive model to identify children with gastrointestinal congenital malformations at high risk of 30-day mortality following intervention or hospital admission.

**Methods:**

The data used for our analysis was collected as part of the Global PaedSurg research collaboration, and includes 3849 patients from 74 countries. Data preprocessing, missing data imputation, oversampling of the non-survivor class, and random undersampling of the survivor class were performed prior to training a random forest classifier for mortality prediction.

**Results:**

The overall 30-day mortality in our model dataset is 19.5%. The model displays an overall accuracy of 88.84% (CI: 86.79%, 90.66%), with strong precision (84.13%, CI: 78.4%, 88.8%) and sensitivity (89.98%, CI: 87.8%, 91.9%) in identifying non-survivors. The area under the curve (AUC) is 0.941 (CI: 0.924, 0.957) for subjects in the non-survival class. The most important features in the classifier are the diagnosis of a complication, the duration of postoperative antibiotic treatment, the need for parenteral nutrition or ventilation, the American Society of Anesthesiologists (ASA) score, weight upon admission, and the use of a surgical safety checklist.

**Conclusions:**

Random forest classifier is a viable option for short-term mortality prediction for children with gastrointestinal congenital malformations.

**Impact:**

- To our knowledge, this is the first global study to apply machine learning for mortality prediction in children with gastrointestinal malformations, using data from 3849 patients across 74 countries.
- The random forest model achieves 88.84% (CI: 86.79%, 90.66%) accuracy, with strong precision (84.13%, CI: 78.4%, 88.8%) and sensitivity (89.98%, CI: 87.8%, 91.9%) in identifying at-risk patients.
- Key predictors include clinical factors (diagnosis of complications, American Society of Anaesthesiologists score, weight on admission, duration of post-operative antibiotic treatment), procedural elements (surgical checklist), and socio-demographic variables (continent, income level).

## Introduction

Global under-5 mortality has steadily declined over recent decades, with a notable 63% reduction, from 12.6 million deaths in 1990 to 4.7 million in 2021.^1^ This progress is largely attributed to advancements in the prevention and treatment of infectious diseases, including measles, malaria, diarrheal diseases, and pneumonia.^2^ Despite these improvements, congenital anomalies remain a leading cause of childhood mortality and long-term disability. Each year, an estimated 240,000 newborns die during the neonatal period, with an additional 170,000 deaths occurring among children aged 1 month to 5 years.^3^

Gastrointestinal congenital defects are associated with particularly high mortality rates, reaching 39.8% in low-income countries, 20.4% in middle-income countries, and 5.6% in high-income countries.^4^ These stark disparities in survival outcomes across healthcare systems with varying resource availability underscore the urgent need for research in this field to better understand the factors influencing mortality.

Our study uses demographic, clinical, diagnostic, intervention, and outcome data from patients with gastrointestinal congenital malformations across 264 hospitals in 74 countries. In an era of data overload, we adopt a practical approach to highlight the value of the secondary use of medical records. Leveraging the publicly available dataset originally collected by us and collaborators worldwide as part of the Global PaedSurg Collaboration,^5,6^ we build a machine learning (ML) algorithm capable of predicting in-hospital survival with enhanced accuracy. Complementing prior research in the field, our random forest (RF) classifier outperforms classical approaches by capturing non-linear relationships and interactions among the variables analyzed. Beyond its predictive value, the model serves as a clinically meaningful tool to support early risk stratification and inform care decisions. More broadly, given the generally low prevalence of high-mortality conditions in pediatrics, this study serves as a proof of concept for a transferable methodology that may be applied to a range of critical conditions impacting children’s survival and well-being.

Traditionally, logistic regression has been the statistical approach used for modeling the probability of binary outcomes, such as survival or mortality.

However, a major progress in mortality prediction analysis was made in the early 2000s, with the adoption of ML techniques in healthcare research. One of the earliest algorithms used, which remains a cornerstone of ML in predictive analysis, is RF. RF has been successfully applied across a range of medical applications, showing superior results when compared to other ML techniques, such as 1-nearest neighbor, AdaBoost (AD), support vector machine, RBF network, and multilayer perceptron, particularly in terms of accuracy, robustness, and handling of high-dimensional data.^7–9^

An RF is an ensemble learning method, i.e., based on multiple decision trees, each trained on a bootstrap sample of the data with a random subset of features. In contrast with single classifiers, which base predictions on a single sequence of splits, RF’s final prediction is made by aggregating the predictions from all individual trees. This is typically done through majority voting in the case of classification tasks.^9^

RF algorithms present several compelling advantages that make them widely applicable across diverse fields. Firstly, as non-parametric models, RFs do not assume a predetermined data distribution, making them robust and versatile for handling data with complex, non-linear relationships. They achieve high classification accuracy by aggregating the predictions of multiple decision trees, effectively reducing variance and mitigating overfitting—a common challenge in traditional single-tree models. Additionally, RF models offer a mechanism to assess variable importance, enabling researchers to identify and rank the most influential features within large datasets, which is particularly valuable in fields such as bioinformatics, financial modeling, and image recognition. Despite these strengths, RFs are often considered “black-box” classifiers because the individual decision rules within each tree remain obscured, preventing straightforward interpretation of how specific input variables contribute to each decision. This limitation can hinder the model’s transparency, especially in critical applications where interpretability is essential. Nevertheless, RFs incorporate a robust algorithm for estimating and imputing missing values, allowing for more complete data utilization and enhanced predictive accuracy. Furthermore, RFs are not restricted to a single analysis type; their flexible framework accommodates a range of analytic tasks, including regression, classification, unsupervised learning, and survival analysis.^10,11^

The objective of our study is to build a clinically valuable survival prediction tool for children with gastrointestinal congenital malformations by using an RF algorithm. In order to reach this objective, we aim to address the following research questions (RQ):

*RQ1:* Can a random forest model accurately predict 30-day survival following intervention or hospital admission in children with gastrointestinal congenital malformations?

*RQ2:* What are the most significant predictors of 30-day survival following intervention or hospital admission in children with gastrointestinal congenital malformations, based on a random forest classifier?

To our knowledge, this is the first study to employ an ML algorithm for predicting survival outcomes in children with gastrointestinal malformations. This innovative approach not only fills a critical gap in pediatric prognosis research but also demonstrates the potential of ML to enhance predictive accuracy and inform clinical decision-making for rare, high-risk conditions. Accurate predictions of higher short-term mortality risk, as well as identifying factors related to such an increased risk, will enable clinicians to tailor clinical care in order to optimize chances of survival for their patients, as well as inform decision-making processes in healthcare policy design. Importantly, this research underscores the value of secondary health data use, transforming existing data into actionable insights and maximizing research impact. By leveraging these data for predictive modeling, our study aligns with the World Health Organization’s goals of advancing data-driven healthcare and promoting sustainable research practices that drive improved health outcomes globally.^12^

Our study is a complementary advancement of prior knowledge in the field. The penalized Lasso regression model used in the initial Global PaedSurg data analysis was very useful in providing risk ratios (RR) of mortality for each variable in the model, thus identifying significant linear associations between clinical and socioeconomic factors and mortality.^4^ Our RF model advances this work by uncovering non-linear interactions and providing robust feature importance rankings to refine risk prediction. Through advanced preprocessing techniques, including imputation of missing data and class imbalance correction, our study ensures robust and generalizable predictions, expanding on the statistical methods used in previous research. Additionally, our study builds on the linear relationships highlighted in the Lasso regression analysis by quantifying the relative importance of variables within a predictive framework, thus enabling targeted policy recommendations.

## Methods

### Study design and cohort description

The data used for our analysis was collected by our team and others as part of the Global PaedSurg research Collaboration, and extensive information on this project has been published, detailing both the research protocol and the study results.^4,5^ The dataset is publicly available, being hosted on the Open Science Framework.^6^

The research is focused on the most common congenital gastrointestinal conditions, including esophageal atresia, congenital diaphragmatic hernia (CDH), intestinal atresia, gastroschisis, exomphalos, anorectal malformation, and Hirschsprung’s disease. The study population comprises children from the newborn stage up to 16 years of age presenting for the first time to the hospital, with one or more specified conditions requiring intervention. Eligible patients received one of the three care options: primary surgical treatment, conservative management, or palliative care. Exclusion criteria included elective admissions, a history of prior surgery for the same condition, presentations related to postoperative complications, as well as transfers to other facilities for surgical intervention. Patients from low-income, middle-income, and high-income countries were included, based on the World Bank country income status classification.^4,13^

The variables collected in the database included demographic information, information regarding antenatal investigations and prehospital care, clinical care, as well as details regarding the type of intervention and outcomes. The primary outcome measured in the study was all-cause in-hospital mortality, stratified by country income status, over a period of 30 days post-intervention if patients underwent an intervention, or 30 days post-admission otherwise.^5^

From an ethical perspective, this study was designed to ensure that patient care was not influenced in any way by participation. All data collected were fully anonymized, protecting patient confidentiality throughout the research process. The study was classified as a clinical audit at the host institution, which, following institutional guidelines, did not require formal ethical approval. However, each participating institution worldwide obtained ethical approval or exemption in line with their local regulatory requirements, ensuring compliance with relevant ethical standards across all regions involved.^5^

### Dataset preparation for the random forest algorithm

The initial dataset collected included 156 variables.^5^ In order to build our RF classifier, additional data cleaning and feature engineering were performed.

In a preliminary step of data cleaning, variables related to administrative aspects, rather than clinical characteristics, were removed (record ID, month of data collection, as well as data referring to the informed consent). Variables related to the cause of death were also excluded from the model, as analyses involving these variables were deemed beyond the scope of the current study (*cause_of_death, other_cause_of_death*). Moreover, the variable selection targeted the general prediction factors, rather than condition-specific variables, in order to increase the generalizability of our findings to the wider context of congenital malformations. We then evaluated issues such as collinearity or a very high volume of missing data. Variables with over 25% missing data were removed from the final database used for the classification algorithm (Table S1, Supplementary Material).

In alignment with our study approach, the outcome variable was *survival*, a binary variable where a value of 1 indicated that the patient was alive in the hospital 30 days after primary intervention or 30 days after presentation in patients who did not receive a primary intervention, and a value of 0 if the patient died in the hospital during the 30-day period after primary intervention or 30 days after presentation in patients who did not receive a primary intervention. Patients with missing or incomplete follow-up data after discharge, resulting in unavailable primary outcome information, were excluded from the final analysis (*n* = 290; 10.2%).

In order to enhance the interpretability and predictive value of the analysis, feature engineering was performed. Variables evaluating hypovolemic or hypothermic status upon admission were combined into one single variable due to collinearity. This variable, *hypother_or_hypovol* was attributed the value “Yes” when either one of the two initial variables had the value “Yes,” and “No” otherwise. Additionally, an aggregated variable, *other_anomalies*, was constructed by combining eleven binary indicators of specific associated anomaly types. Another composite variable, *complic_diagnosed*, was created to capture postoperative complications by aggregating data from surgical site infection (*ssi*), wound dehiscence (*wound_dehis*), and the need for further intervention (*further_intervention*). To facilitate stratified analyses by treatment modality, the variable capturing time to surgical intervention (*time_interv*) was coded as NA for patients managed exclusively with medical therapy. A new binary variable, *treatment_type*, was created to indicate whether a patient underwent at least one surgical procedure or received exclusively medical treatment.

The definitions of all the variables included in the RF model setup, based on the primary Global PaedSurg database,^6^ are presented in Table S2, in the Supplementary Material.

After the general preparation of the dataset for the RF classification algorithm, we continued preprocessing through dividing eligible patients into a training and a validation set, in a 7:3 ratio, using stratified sampling, based on the country’s income category (low-, middle-, or high-income).

Moving forward, we performed missing data imputation. For this purpose, we performed a comparative evaluation of the performance of two widely used methods for data imputation, the rfImpute function from the *randomForest* package, and the missForest function from the homonymic package. The two functions have different approaches. On the one hand, *rfImpute* uses class-specific proximities derived from an initial RF and imputes missing values by proximity-weighted averaging. In contrast, *missForest* starts by filling in missing values with simple initial guesses (e.g., means for numeric variables), then fits an RF model for each variable with missing data, using the other variables as predictors. The algorithm iteratively updates the missing values until convergence or a stopping rule is met.^14^ In our study, for the train set, the rfImpute had high overall out-of-bag classification error rates (10.67%–11.15% over 5 iterations) and larger class-specific errors (46.96%–48.38% for non-survivors and 1.7%–1.9% for survivors). However, missForest provided high stability and balance in imputation quality. The estimated normalized root mean squared error for continuous variables ranged between 0.4941 and 0.5307, indicating moderate imputation accuracy. Across iterations, the differences between successive imputed matrices decreased steadily (as low as 2.85e-05), suggesting convergence. No error was reported for categorical variables, indicating perfect classification consistency during imputation. Based on its robust performance and reliable convergence across iterations, missForest was selected for missing data imputation in the training dataset.

Missing data in the validation dataset were imputed separately using summary statistics derived from the training set. Specifically, we avoided reapplying the imputation model directly to the validation data to prevent data leakage. Instead, missing numeric values in the validation dataset were imputed using the median of the corresponding variable from the training data, while missing categorical values were imputed using the most frequent category, i.e, the mode, observed in the training data.

Given the significant over-representation in the sample of patients with short-term survival, compared to those who deceased in the first 30 days post-intervention or admission to the hospital, we also explored methods of addressing class imbalance. In particular, we have chosen to focus on data-level techniques, oversampling of the non-survivor class, undersampling of the survivor class, as well as a hybrid approach, combining both. This step was crucial in order to mitigate model bias toward the majority class and enhance predictive performance for the minority class.^15^ Resampling was performed prior to model training, producing a balanced training set with an approximately 1:1 class ratio. The final resampled train dataset included 2494 patients, comprising 1247 survivors and 1247 non-survivors.

Finally, we used the randomForest function in our *train* dataset in order to implement Breiman’s RF classification algorithm.^10,16^

### Classifier performance evaluation

In order to evaluate the performance of our model in the validation dataset, we first used a confusion matrix. This allowed for the calculation of accuracy, sensitivity, specificity, and F1 score, by comparing actual values in the validation dataset and values assigned by the RF classifier.^17^

Additionally, in order to gain a deeper understanding of the predictive capabilities of our algorithm, we used the predict function, applied with the argument type = “prob.” By instructing the model to return class probabilities and appending [,2], the code extracted the probabilities corresponding to the second class. The “positive” or minority class was represented in our case by patients who did not survive 30 days post-intervention or admission. We then generated a receiver operating characteristic (ROC) curve, using the roc function from the *pROC* package, and calculated the area under the curve (AUC), using the auc function.^18,19^ By comparing true binary outcomes, the survival status in our case, with the predicted probabilities for the survivor class, we were able to quantify the model’s ability to distinguish between the two classes, with values closer to 1 indicating better performance of the RF model.

For the next step of our analysis, we identified, based on our optimized RF classifier, the most important determinants of short-term mortality prediction in children with gastrointestinal congenital malformations. To this purpose, we used the FeatureImp function in the *IML* package. The basic principle of this method relies on measuring model performance drops subsequent to shuffling each feature included in the classifier.^20^ Feature selection aligns with the aim of our study, allowing for increased generalizability by extracting the most informative features for mortality prediction.^21^

All analyses included in this research were conducted using the R programming language (version 2021.09.0; R Core Team). Key packages used include *dplyr* for data manipulation,^22^ *randomForest*^16^ and *missForest*^14^ for imputation and modeling, *pROC*^19^ and *ROCR*^23^ for performance evaluation, and *caret*^24^ for model training and validation workflows.

## Results

For our RF classifier, patient inclusion was based on the availability of survival outcomes and complete follow-up data. A cohort of 3559 patients was extracted from the original Global PaedSurg database, with 3680 study conditions, among which 524 were patients with esophageal atresia, 420 had CDH, 640 had intestinal atresia, 417 had gastroschisis, 297 had exomphalos, 907 had anorectal malformation, and 475 had been diagnosed with Hirschsprung’s disease. Surgical intervention was the primary treatment modality for most patients (*n* = 3187; 89.6%).

In predictive modeling, the composition of the training dataset plays a critical role in determining model performance. When analyzing mortality, a disproportionate number of survivors can bias the model toward the majority class, i.e., overestimate the chances of survival for a patient. Our dataset also exhibited a significant class imbalance, with survivors notably overrepresented. Table 1 provides a breakdown of class distribution across each dataset.

**Table 1:** Distribution of data in the training and test datasets

Dataset	Classes	Short-term survival
Train	No (non-survivors)	485
	Yes (survivors)	2006
Test	No (non-survivors)	208
	Yes (survivors)	860
Total	No (non-survivors)	693 (19.47%)
Total	Yes (survivors)	2866 (80.53%)

Important imbalance between the minority class—non-survivors and the majority class—survivors.

In this context, data balancing was of paramount importance in improving the performance of our model and its accuracy in predicting mortality in different clinical scenarios. Table 2 contains a comparative overview of the confusion matrix and main associated metrics obtained for our RF model, after using different approaches for balancing the data: a hybrid approach, oversampling of the non-survivors, or undersampling of survivors.^25^

**Table 2:** Confusion matrix metrics comparing data balancing techniques: hybrid resampling achieves optimal predictive performance

Metric	Model A (oversampling of non-survivors and undersampling of survivors)	Model B (oversampling of non-survivors)	Model C (undersampling of survivors)
Accuracy	89.31% (87.29%, 91.10%)	84.6% (82.3%, 86.7%)	84.8% (82.5%, 86.9%)
Sensitivity (detect non-survivors)	77.88% (74.9%, 80.6%)	85.1% (82.5%, 87.4%)	86.5% (84%, 88.7%)
Specificity (detect survivors)	92.07% (87.8%, 95.5%)	84.5% (79%, 89.2%)	84.4% (79%, 89.2%)
F1 score	0.74 (0.692, 0.786)	0.684 (0.638, 0.729)	0.69 (0.642, 0.735)
AUC (area under the curve)	0.941 (0.924, 0.957)	0.939 (0.922, 0.957)	0.940 (0.922, 0.957)

Values are shown with 95% confidence intervals.

In our case, data balancing through a combined approach allowed us to achieve the highest overall accuracy (89.31%, 95% CI: 87.29%–91.10%), the highest specificity (92.07%, 95% CI: 87.8%–95.5%), and the strongest agreement between predicted and true outcomes as indicated by Cohen’s Kappa (0.74, 95% CI: 0.692–0.786).

In order to evaluate the performance of our model, we compare these values to the No Information Rate (NIR), meaning the accuracy of a theoretical prediction always choosing the majority class, i.e., survivors. Based on the composition of our sample, the NIR is 80.52%, significantly lower than our model accuracy (*p* < 0.001), meaning that the model is adding real value compared to the most common outcome.

**Fig. 1:**
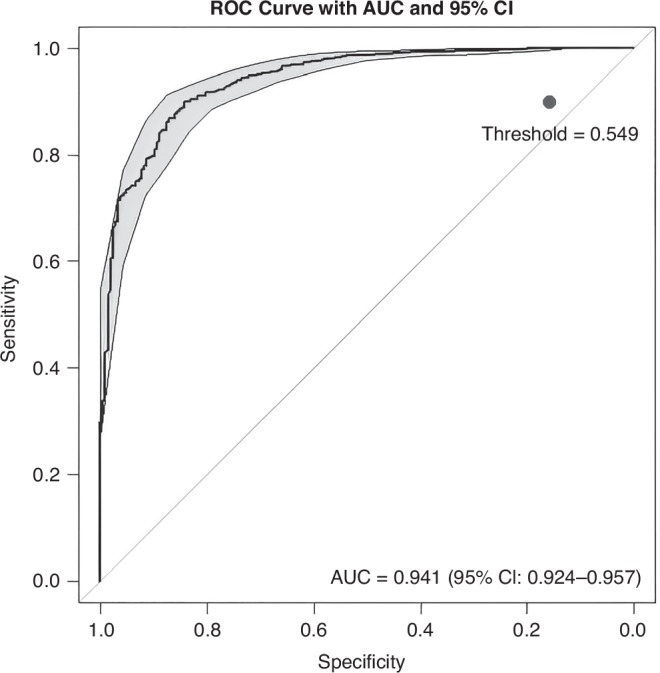
The receiver operating characteristic (ROC) curve evaluates the trade-off between sensitivity and specificity in predicting non-survival. The area under the curve (AUC) indicates excellent discriminatory performance of the Random Forest model for patients who did not survive 30 days post-intervention or admission.

In order to further examine the performance of our model in identifying patients at high risk of non-survival, we evaluated the ROC curve for the non-survivor class. The model displayed an AUC of 0.941 (95% CI: 0.924, 0.957). Given the clinical importance of accurately identifying both survivors and non-survivors, we used the threshold that maximized Youden’s Index.^26^ This approach allowed us to identify the optimal cutoff, 0.549. This threshold was chosen over the default 0.5 to better reflect the trade-off between false negatives and false positives in our survival prediction model (Fig. 1). The final model, based on the adjusted threshold of 0.549, has an accuracy of 88.84% (95% CI: 86.79%, 90.66%), a sensitivity of 89.98% (95% CI: 87.8%, 91.9%), a specificity of 84.13% (95% CI: 78.4%, 88.8%) and an F1 score of 0.746 (95% CI: 0.701, 0.791).

Moreover, to assess the generalizability of the classifier across varying healthcare settings, we evaluated model performance stratified by national income level—low-, middle-, and high-income countries (Supplementary Table S3), as well as by congenital malformation subtype (Supplementary Table S4). While the model demonstrated strong performance across all strata, small sample sizes in some subgroups may have contributed to less stable estimates, underscoring the need for further validation in larger datasets.

**Fig. 2:**
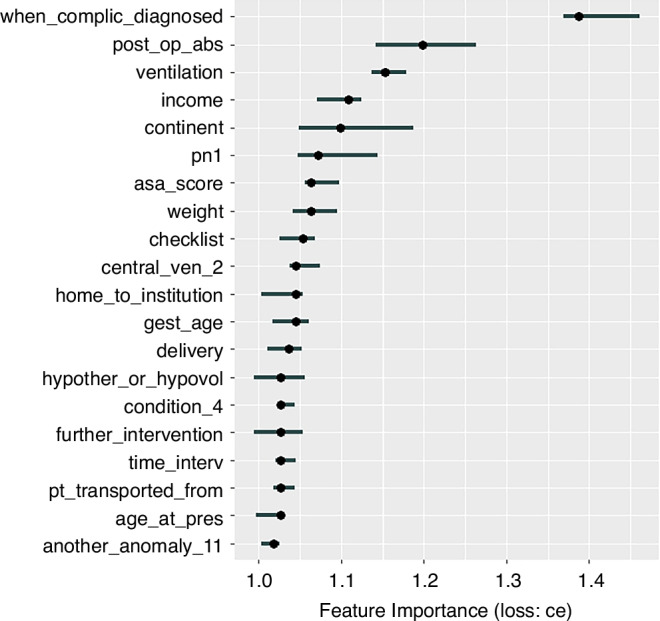
Random forest classifier feature importance analysis. Variable definitions are detailed in Table S2. The most important feature identified in our classifier was the diagnosis of a complication—surgical site infection, full-thickness wound dehiscence, or need for further unplanned re-intervention. This was followed by the duration of postoperative antibiotic treatment, the patient’s requirements for parenteral nutrition and/ or ventilation, as well as the American Society of Anesthesiologists (ASA) score. Socio-demographic factors also have significant importance, including the continent of origin and the country’s income level (low, middle, or high).

In order to address our second research question, we performed feature importance analysis. Results are summarized in Fig. 2. The most important features include the diagnosis of complications, the duration of postoperative antibiotic treatment, the need for ventilation or parenteral nutrition, the American Society of Anaesthesiologists score, patient’s weight on admission, as well as procedural elements (surgical checklist), and socio-demographic variables (continent, country income level).

## Discussion

This research validates an ML algorithm for the prediction of short-term survival, 30 days post-intervention or admission, of children with gastrointestinal congenital malformations. Through the RF feature importance analysis, we are able to identify the main factors influencing mortality prediction, thus building on prior knowledge and enabling targeted policy recommendations.^21^

### Practical implications

When comparing our results to those of the penalized Lasso regression included in the initial Global PaedSurg Study,^5^ we can extract interesting insights that warrant further discussion. Based on RR, the regression analysis identified factors significantly associated with higher mortality, including lower country income status, the presence of sepsis at presentation, higher American Society of Anesthesiologists (ASA) scores at primary intervention, lack of use of a surgical safety checklist, and unavailability of ventilation or parenteral nutrition when required.^5^ In turn, while inherently more complex, the RF classifier offers actionable insights through its feature ranking based on their contribution to predictive accuracy. The RF analysis reinforces the findings from the initial study by confirming the role of socio-demographic factors, including the country-of-origin income level (low, middle, or high), the ASA score, the use of a surgical checklist, the patient’s weight upon admission, as well as the need for parenteral nutrition or ventilation. Our results also show that the early diagnosis of a complication (full-thickness wound dehiscence, surgical site infection, or need for unplanned surgical re-intervention) is a factor contributing significantly to the accuracy of mortality prediction. Compared to the penalized Lasso regression, the RF classifier identifies more specifically the duration of postoperative antibiotic treatment as significantly increasing accuracy in mortality prediction, while the binary evaluation of septic status upon admission plays a significantly smaller role.

The main source of impact for our research stems from its practical utility. This algorithm is a proof-of-concept supporting the use of RF classifiers in clinical scenarios, where they could serve as robust decision-support tools for clinicians, complementing traditional scoring systems like ASA. By allowing for a timely detection of high-risk patients, ML tools could inform therapeutic adjustments for improved clinical outcomes.

### Limitations and future directions

Despite its considerable strengths, our ML approach to mortality prediction has several important limitations. A key concern lies in the heterogeneity of the patient population. While the analysis focuses on gastrointestinal anomalies, several conditions included—such as CDH and omphalocele—are syndromic or involve multiple organ systems, adding complexity to both diagnosis and prognosis. Furthermore, there is substantial variation in the timing of diagnosis, availability of therapeutic resources, and clinical expertise across different geographic regions. Although stratified sampling was employed to ensure balanced geographic representation in both the training and validation datasets, this inherent variability must be acknowledged when considering the generalizability of the model to other settings or patient populations.

Moreover, RF is described as a “black-box” model, meaning its predictions are harder to interpret compared to more traditional approaches, such as the Lasso regression. Clinicians may struggle to understand how specific variables contribute to a patient’s risk score, which could reduce trust in the model. Post-hoc explainability techniques, such as SHAP values or partial dependence plots, could be beneficial in order to make an RF classifier largely usable in a clinical scenario.^27,28^

Secondly, while adaptability is a main trait of RF algorithms, its degree needs to be further assessed based on each individual classifier. In theory, RF models can handle missing data, imbalanced datasets, and a mix of categorical and continuous variables without extensive preprocessing, making them particularly valuable in multi-center datasets with variable data quality. However, it is worth noting that, in our case, this characteristic can be further nuanced with regard to the practical consequences. Various data imputation and class balancing techniques can yield prediction models with markedly different performance metrics. In clinical settings, selecting an appropriate model involves weighing the trade-off between sensitivity and specificity, depending on which type of misclassification—false positives or false negatives—is more critical. For example, in our study, oversampling the non-survivor class and undersampling the survivor class each resulted in models with slightly higher sensitivity (85.1% and 86.5%, respectively) compared to the hybrid method. However, these gains came at the expense of specificity and overall accuracy. Given the clinical consequences of both types of errors, we determined that the hybrid resampling approach offered a more balanced and clinically appropriate solution for addressing class imbalance. The choice of a predictive model in a clinical scenario will depend on the particular context and the goals envisioned. The impact of our research could be further expanded through the use of hybrid strategies that could combine, for example, RF for predictive accuracy and Lasso regression for interpretability. Additionally, other ML approaches could be explored, such as gradient boosting machines or neural networks.

## Conclusion

The RF model demonstrated high accuracy and strong discriminatory power, particularly in identifying survivors and non-survivors following surgical intervention or medical management. These findings support the model’s potential utility in aiding clinical decision-making in scenarios involving high-risk patients. Its adjuvant role to clinical reasoning can be of paramount importance in the context of gastrointestinal congenital anomalies. Our findings underscore the critical need to prioritize neonatal surgical care within the global child health agenda to achieve Sustainable Development Goal 3.2—ending preventable deaths in neonates and children under five by 2030. A comprehensive health systems strengthening approach is essential, addressing all levels of care and involving the entire multidisciplinary team. Key priorities include: (1) improving antenatal diagnosis and ensuring delivery at centers equipped for pediatric surgery; (2) enhancing early resuscitation at district hospitals and safe, timely transfer to surgical centers; and (3) optimizing perioperative care within specialist facilities.

Given the high mortality rate associated with gastrointestinal congenital anomalies,^4^ as well as the paucity of large-scale studies in the field, our ML prediction algorithm, based on the largest international cohort to date, is a valuable tool for pediatric surgical teams worldwide. Moreover, our research highlights the importance of utilizing secondary health data, amplifying the research impact of existing datasets and complementing prior knowledge with additional actionable insights.

## Supplementary information

Supplementary Material

Supplementary Material

Supplementary Material

Supplementary Material

## Data Availability

The original dataset used for this article is freely available on the Open Science Framework database and can be consulted here.

## References

[1] GBD Results. Institute for Health Metrics and Evaluation. https://vizhub.healthdata.org/gbd-results.

[2] GBD 2015 Child Mortality Collaborators. Global, regional, national, and selected subnational levels of stillbirths, neonatal, infant, and under-5 mortality, 1980-2015: a systematic analysis for the Global Burden of Disease Study 2015. *Lancet* **388**, 1725–1774 (2016). 10.1016/S0140-6736(16)31575-6 PMC5224696 27733285

[3] Congenital disorders. https://www.who.int/news-room/fact-sheets/detail/birth-defects.

[4] Wright, N. J. et al. Mortality from gastrointestinal congenital anomalies at 264 hospitals in 74 low-income, middle-income, and high-income countries: a multicentre, international, prospective cohort study. *Lancet* **398**, 325–339 (2021). 34270932 10.1016/S0140-6736(21)00767-4 PMC8314066

[5] Wright, N. J. & Global PaedSurg Research Collaboration. Management and outcomes of gastrointestinal congenital anomalies in low, middle and high income countries: protocol for a multicentre, international, prospective cohort study. *BMJ Open* **9**, e030452 (2019). 10.1136/bmjopen-2019-030452 PMC6731898 31481373

[6] Wright, N. Global PaedSurg Study. https://osf.io/e4uvh/.

[7] Montazeri, M., Montazeri, M., Montazeri, M. & Beigzadeh, A. Machine learning models in breast cancer survival prediction. *Technol. Health Care* **24**, 31–42 (2016). 26409558 10.3233/THC-151071

[8] Woźniacki, A., Książek, W. & Mrowczyk, P. A novel approach for predicting the survival of colorectal cancer patients using machine learning techniques and advanced parameter optimization methods. *Cancers* **16**, 3205 (2024). 39335174 10.3390/cancers16183205 PMC11430446

[9] Alam, Md. Z., Rahman, M. S. & Rahman, M. S. A random forest based predictor for medical data classification using feature ranking. *Inform. Med. Unlocked* **15**, 100180 (2019).

[10] Breiman, L. Random Forests. *Mach. Learn.* **45**, 5–32 (2001).

[11] Rodriguez-Galiano, V. F., Ghimire, B., Rogan, J., Chica-Olmo, M. & Rigol-Sanchez, J. P. An assessment of the effectiveness of a random forest classifier for land-cover classification. *ISPRS J. Photogramm. Remote Sens.* **67**, 93–104 (2012).

[12] Improving health-care delivery and innovation through secondary use of health data. https://www.who.int/europe/activities/improving-health-care-delivery-and-innovation-through-secondary-use-of-health-data.

[13] World Bank Country and Lending Groups—World Bank Data Help Desk. https://datahelpdesk.worldbank.org/knowledgebase/articles/906519-world-bank-country-and-lending-groups.

[14] Stekhoven, D. J. & Bühlmann, P. MissForest—non-parametric missing value imputation for mixed-type data. *Bioinformatics* **28**, 112–118 (2012). 22039212 10.1093/bioinformatics/btr597

[15] Mohammed, R., Rawashdeh, J. & Abdullah, M. Machine learning with oversampling and undersampling techniques: overview study and experimental results. In *Proc. 2020 11th International Conference on Information and Communication Systems (ICICS)* 243–248. 10.1109/ICICS49469.2020.239556 (2020).

[16] randomForest function—RDocumentation. https://www.rdocumentation.org/packages/randomForest/versions/4.7-1.2/topics/randomForest.

[17] Japkowicz, N. & Shah, M. Performance Evaluation in Machine Learning. in *Machine Learning in Radiation Oncology: Theory and Applications* (eds El Naqa, I., Li, R. & Murphy, M. J.) 41–56. 10.1007/978-3-319-18305-3_4 (Springer International Publishing, 2015).

[18] Sachs, M. C. About ROC curves. https://cran.r-project.org/web/packages/plotROC/vignettes/examples.html (2023).

[19] Robin, X. et al. pROC: an open-source package for R and S+ to analyze and compare ROC curves. *BMC Bioinform.* **12**, 77 (2011). 10.1186/1471-2105-12-77 PMC3068975 21414208

[20] Introduction to iml: Interpretable Machine Learning in R. https://cran.r-project.org/web/packages/iml/vignettes/intro.html.

[21] Pudjihartono, N., Fadason, T., Kempa-Liehr, A. W. & O’Sullivan, J. M. A review of feature selection methods for machine learning-based disease risk prediction. *Front. Bioinform.* **2**, 927312 (2022). 36304293 10.3389/fbinf.2022.927312 PMC9580915

[22] A grammar of data manipulation. https://dplyr.tidyverse.org/.

[23] Sing, T., Sander, O., Beerenwinkel, N. & Lengauer, T. ROCR: visualizing classifier performance in R. *Bioinformatics* **21**, 3940–3941 (2005). 16096348 10.1093/bioinformatics/bti623

[24] Kuhn, M. Building predictive models in R using the caret package. *J. Stat. Softw.* **28**, 1–26 (2008).27774042

[25] Hicks, S. A. et al. On evaluation metrics for medical applications of artificial intelligence. *Sci. Rep.* **12**, 5979 (2022). 35395867 10.1038/s41598-022-09954-8 PMC8993826

[26] Fawcett, T. An introduction to ROC analysis. *Pattern Recognit. Lett.* **27**, 861–874 (2006).

[27] Song, S. I., Hong, H. T., Lee, C. & Lee, S. B. A machine learning approach for predicting suicidal ideation in post stroke patients. *Sci. Rep.* **12**, 15906 (2022). 36151132 10.1038/s41598-022-19828-8 PMC9508242

[28] Wu, L. et al. A machine learning model for predicting prognosis in HCC patients with diabetes after TACE. *J. Hepatocell. Carcinoma* **12**, 77–91 (2025). 39867262 10.2147/JHC.S496481 PMC11762020

