# Supplementary material for: Short-term mortality prediction in children with gastrointestinal congenital anomalies using a random forest classifier
Source: Pediatr Res. 2025 Sep 15;99(3):909–14. doi: 10.1038/s41390-025-04378-2 (PMC13021500; doi:10.1038/s41390-025-04378-2)
Supplement: Supplementary file 1 — Supplementary Material [file 41390_2025_4378_MOESM1_ESM.pdf]

Annex 1. Supplementary Material

Table S1. Variables Eliminated Prior to Model Training Due to Incomplete Data (25% missing data threshold)

No.	Variable Name	Item Description
1	dx_ga	Gestational age at the time of the anatenatal diagnosis
2	appro_abx	Use of appropriate antibiotics for septic patients based on bacteria sensitivity
3	iv_given	Binary variable evaluating the administration of an intravenous fluid bolus to hypovolemic patients
4	ivf_volume	Volume of fluid bolus administered to hypovolemic patients
5	vent_days	Number of days a patient remained on ventilation
6	time_to_first_feed	Number of days to first enteral feed
7	time_to_full_feeds	Number of days to full enteral feeds
8	pn_days	Number of days of parenteral nutrition
9	duration_hosp_stay	Number of days of hospital stay
