# Supplementary material for: Short-term mortality prediction in children with gastrointestinal congenital anomalies using a random forest classifier
Source: Pediatr Res. 2025 Sep 15;99(3):909–14. doi: 10.1038/s41390-025-04378-2 (PMC13021500; doi:10.1038/s41390-025-04378-2)
Supplement: Supplementary file 3 — Supplementary Material [file 41390_2025_4378_MOESM3_ESM.pdf]

Table S3. Model Performance Evaluation by Country Income Status based on the World Bank Country Classification:

HIC- High Income Countries, MIC- Middle Income Countries, LIC- Low Income Countries

**Metric**	**HIC** (n=249)	**MIC** (n=796)	**LIC** (n=21)
**Accuracy**	95.58% (91.6%–98.7%)	87.56% (87.1%–92.1%)	80.95% (58.1%–94.6%)
**Sensitivity (detect non-survivors)**	57.1% (34.0%–78.2%)	79.9% (73.3%–85.5%)	100% (63%-100%)
**Specificity (detect survivors)**	99.1% (96.9%–99.9%)	89.8% (87.1%–92.1%)	69.23% (38.6% – 90.9%)
**F1 score**	0.64 (0.444-0.798)	0.841 (0.812-0.866)	0.792 (0.571-0.957)
**AUC (Area Under the Curve)**	0.95 (0.92–0.99)	0.94 (0.92–0.96)	0.89 (0.76–1.00)

Values are shown with 95% confidence intervals.
