# Supplementary material for: Short-term mortality prediction in children with gastrointestinal congenital anomalies using a random forest classifier
Source: Pediatr Res. 2025 Sep 15;99(3):909–14. doi: 10.1038/s41390-025-04378-2 (PMC13021500; doi:10.1038/s41390-025-04378-2)
Supplement: Supplementary file 4 — Supplementary Material [file 41390_2025_4378_MOESM4_ESM.pdf]

Table S4. Model Performance Evaluation by Congenital Malformation Subtype

Condition	Number of patients	Accuracy	Sensitivity	Specificity	F1 Score	AUC
**Oesophageal atresia**	149	0.866 (0.800–0.916)	0.750 (0.578–0.879)	0.903 (0.832–0.950)	0.698 (0.538–0.822)	0.921 (0.864–0.979)
**CDH**	131	0.863 (0.792–0.916)	0.773 (0.622–0.885)	0.908 (0.827–0.959)	0.730 (0.598–0.840)	0.927 (0.881–0.973)
**Intestinal atresia**	193	0.860 (0.803–0.906)	0.744 (0.588–0.865)	0.893 (0.833–0.938)	0.709 (0.579–0.815)	0.921 (0.881–0.960)
**Gastroschisis**	122	0.934 (0.875–0.971)	0.878 (0.738–0.959)	0.963 (0.896–0.992)	0.860 (0.744–0.934)	0.983 (0.966–1.000)
**Exomphalos/ Omphalocele**	92	0.880 (0.796–0.939)	0.722 (0.465–0.903)	0.919 (0.832–0.970)	0.680 (0.462–0.839)	0.908 (0.839–0.978)
**ARM**	279	0.910 (0.871–0.941)	0.880 (0.688–0.975)	0.913 (0.872–0.945)	0.787 (0.658–0.879)	0.946 (0.899–0.993)
**Hirschsprung disease**	142	0.930 (0.874–0.966)	0.500 (0.187–0.813)	0.962 (0.914–0.988)	0.444 (0.196–0.713)	0.919 (0.841–0.998)

Values are shown with 95% confidence intervals.
